# Diffusion-controlled bridging of the Au Island and Au core in Au@Rh(OH)_3_ core-shell structure

**DOI:** 10.3389/fchem.2023.1138932

**Published:** 2023-01-25

**Authors:** Jie Zhang, Quan Ren, Yun Wang, Ruixue Xiao, Hongyu Chen, Wenjia Xu, Yuhua Feng

**Affiliations:** ^1^ Institute of Advanced Synthesis, School of Chemistry and Molecular Engineering, Nanjing Tech University, Nanjing, China; ^2^ School of Science, Westlake University, Hangzhou, China; ^3^ School of Physical and Mathematical Science, Nanjing Tech University, Nanjing, China

**Keywords:** core-shell, plasmonic structure, LSPR coupling, spacing layer, SERS

## Abstract

Hybrid nanostructures have garnered considerable interest because of their fascinating properties owing to the hybridization of materials and their structural varieties. In this study, we report the synthesis of [Au@Rh(OH)_3_]-Au island heterostructures using a seed-mediated sequential growth method. Through the thiol ligand-mediated interfacial energy, Au@Rh(OH)_3_ core-shell structures with varying shell thicknesses were successfully obtained. On these Au@Rh(OH)_3_ core-shell seeds, by modulating the diffusion of HAuCl_4_ in the porous Rh(OH)_3_ shell, site-specific growth of Au islands on the inner Au core or on the surface of the outer Rh(OH)_3_ shell was successfully achieved. Consequently, two types of distinct structures, the Au island-on-[Au@Rh(OH)_3_] dimer and Au island-Au bridge-[Au@Rh(OH)_3_] dumbbell structures with thin necks were obtained. Further modulations of the growth kinetics led to the formation of Au plate-Au bridge-[Au@Rh(OH)_3_] heterostructures with larger structural anisotropy. The flexible structural variations were demonstrated to be an effective means of modulating the plasmonic properties; the Au–Au heterostructures exhibited tunable localized surface plasmon resonance in the visible-near-infrared spectral region and can be used as surface-enhanced Raman scattering (SERS) substrates capable of emitting strong SERS signals. This diffusion-controlled growth of Au bridges in the Rh(OH)_3_ shells (penetrating growth) is an interesting new approach for structural control, which enriches the tool box for colloidal nanosynthesis. This advancement in structural control is expected to create new approaches for colloidal synthesis of sophisticated nanomaterials, and eventually enable their extensive applications in various fields.

## Graphical Abstract

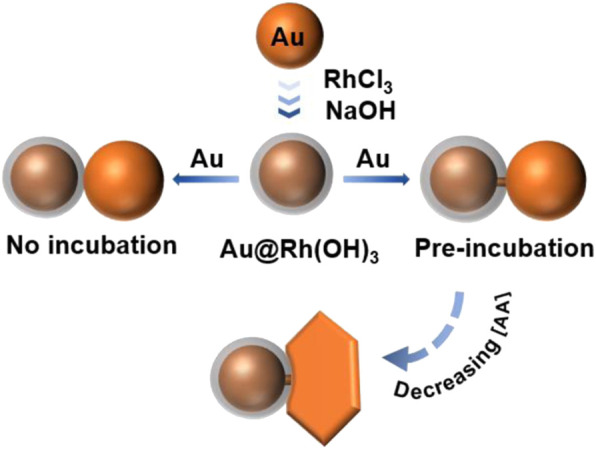

## 1 Introduction

The growth of crystals in a structure inevitably occupies the internal space (Li et al., 2009; Crossland et al., 2013; Shen et al., 2018). When the growth reaches a certain level, it penetrates the material and grows outside the structure. The penetration phenomenon originating from the mechanical force of the crystal growth is common in nature. For instance, in biomineralization (Tampieri et al., 2011; Campodoni et al., 2018; Chen et al., 2019), the growth of biominerals can be divided into two stages: the 1st stage; growth in the tissues, and the 2nd stage; growth outside after penetrating the interface. In lithium batteries, the layered growth of Li can transform into dendrites, and their overgrowth may penetrate the battery membrane, resulting in a short circuit or destruction of the battery structure (Bai et al., 2018; Wang et al., 2019b; Wang et al., 2021; Yang et al., 2022).

The nature of the above phenomena is the overgrowth of new domains at the nanoscale, which eventually leads to the penetration of the interface. In contrast to the result under an applied force, growth-induced penetration is a synergistic effect controlled by multiple factors. Therefore, understanding the laws and mechanisms of growth penetration would be helpful in studying these macroscopic phenomena.

The main challenge of this type of study is the difficulty in controlling the initial growth site and characterizing the growth process in detail. Thus, a suitable model system is very significant in the study of the growth penetration phenomena.

In our previous study, the growth of Ag on the surface of the Ag seeds in the Poly (styrene-b-acrylic acid) (PSPAA) shell (Jiang et al., 2018) was achieved. The continuous extrusion of the PSPAA polymer with Ag growth eventually led to the penetration of the polymer shell. The growth of the Ag outside the polymer shell led to the wrapping of seeds by the Ag nanoplate with a long and narrow nanogap. We speculate that the penetration of the Ag bridge is due to the porous nature of the PSPAA shell after the swelling-deswelling process. The growth of Ag continuously occupied the pores in the PSPAA and eventually penetrated out. Because of the mobility of the PSPAA shell, it is difficult to study the penetration of the PSPAA shell owing to the overgrowth of Ag in the shell.

**FIGURE 1:**
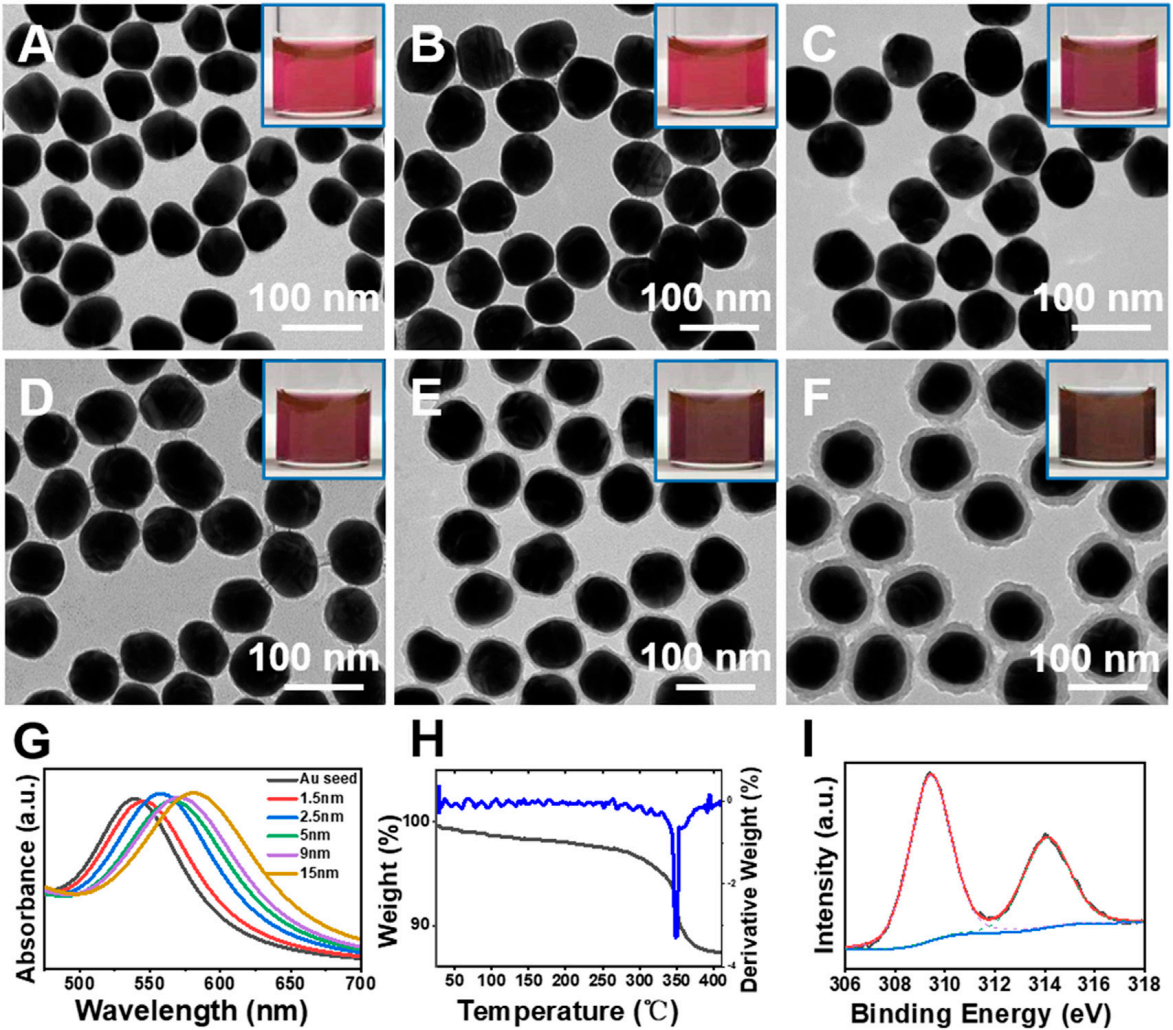
**(A**–**F)** TEM images of Au seeds and Au@Rh(OH)_3_ core-shell structures with varying shell thickness. **(G)** Absorption, **(H)** TG and DTG curves, and **(I)** XPS spectra of the Au@Rh(OH)_3_ structures.

**FIGURE 2:**
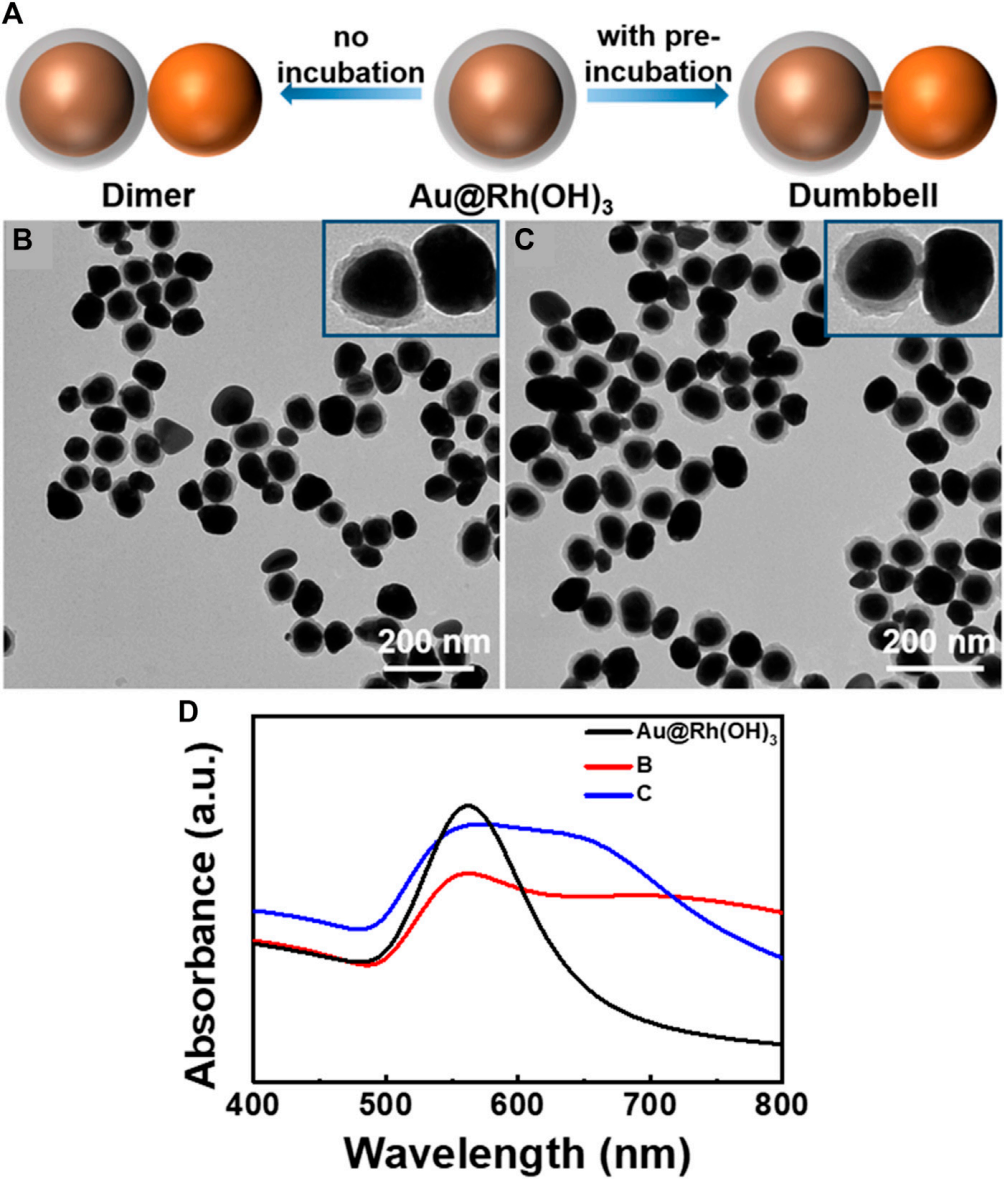
**(A)** Schematics illustrating the synthesis of Au-[Au@Rh(OH)_3_] hetero-structures. TEM images of **(B)** Au island-on-[Au@Rh(OH)_3_] dimer and **(C)** Au island-Au bridge-[Au@Rh(OH)_3_] dumbbell structures. **(D)** Absorption spectra for the structures illustrated in Figures 2B, C.

**FIGURE 3:**
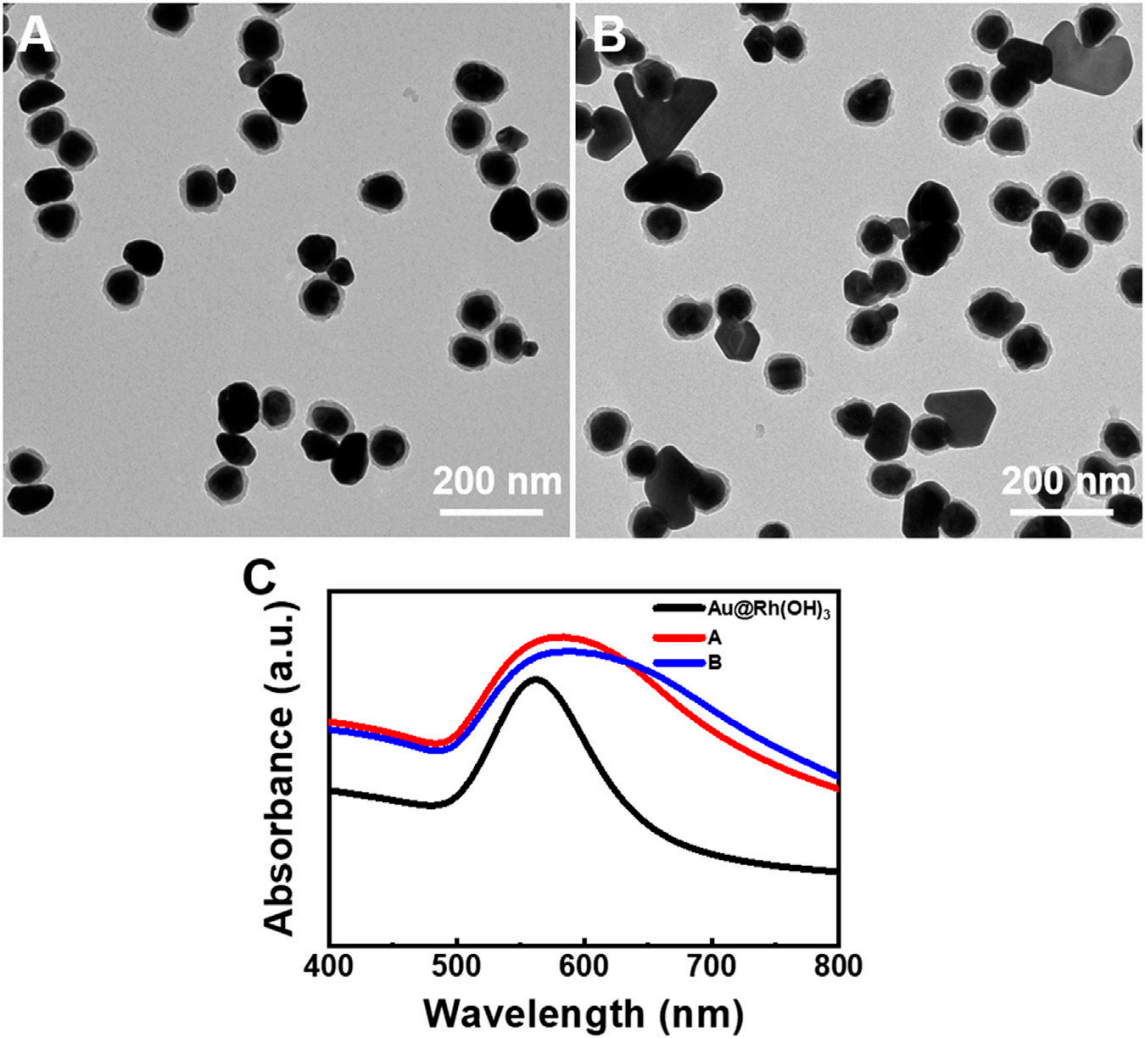
Au–Au@Rh(OH)_3_ dimers synthesized under low AA concentration: **(A)** Without and **(B)** with pre-incubation of Au@Rh(OH)_3_. **(C)** Absorption spectra of the structures illustrated in Figures 3A, B.

**FIGURE 4:**
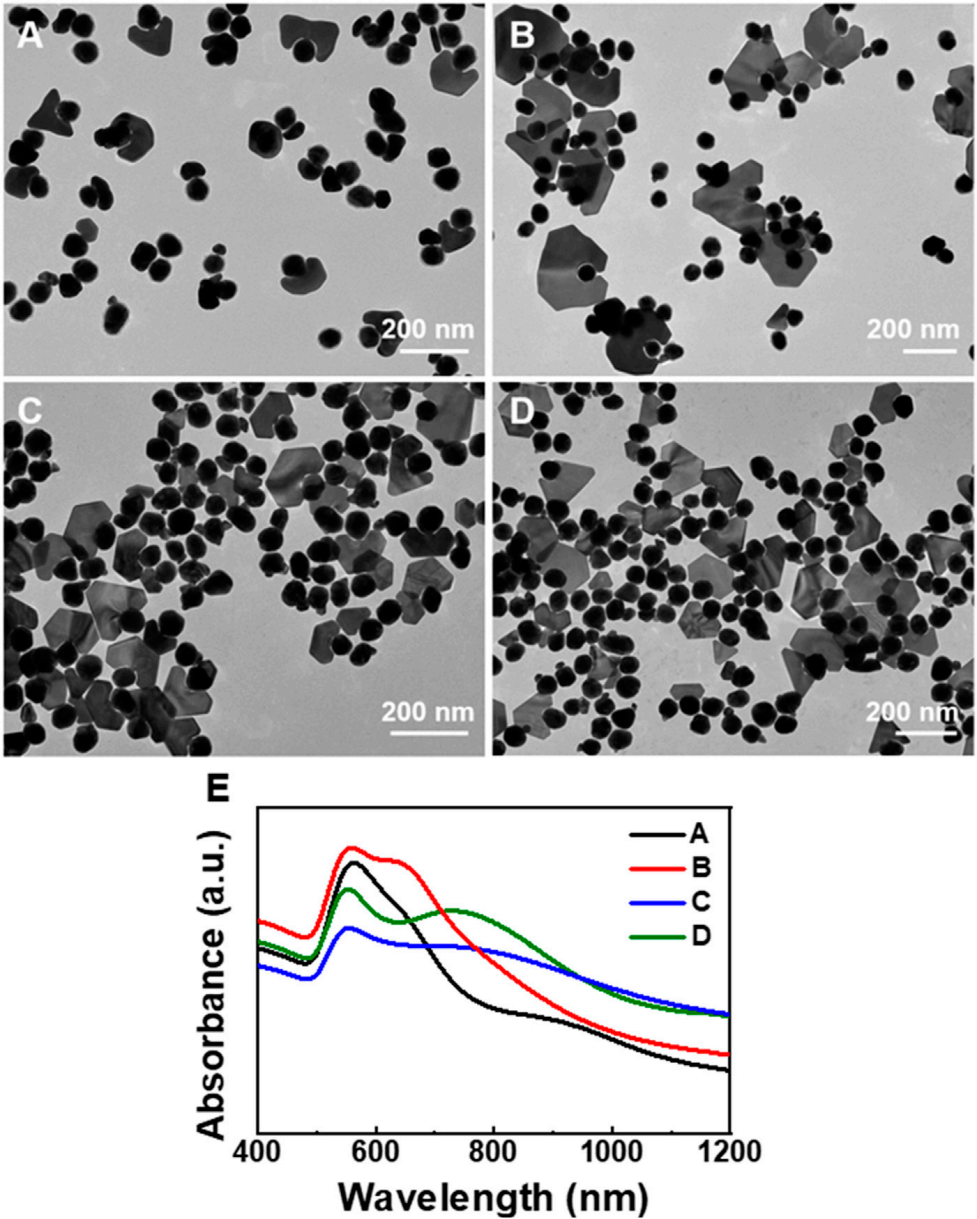
TEM images of the Au plate-Au bridge-[Au@Rh(OH)_3_] structures synthesized using Au@Rh(OH)_3_ seeds with **(A, B)** 8, **(C)** 5, and **(D)** 2.5 nm shell thickness. **(E)** Absorption spectra of the structures illustrated in Figures 4A–D.

Herein, we report that the penetration growth of Au was achieved in the hard porous Rh(OH)_3_ layer in Au@Rh(OH)_3_ core-shell structures. Specifically, through interfacial energy control, uniform concentric Au@Rh(OH)_3_ core-shell structures with varying shell thicknesses were successfully synthesized (Figure 1) and used as seeds for the growth of Au–Au heterostructures. Controlling the diffusion of the HAuCl_4_ precursor in the Rh(OH)_3_ shells led to the formation of two types of Au–Au hybrids: Au island-on-[Au@Rh(OH)_3_] dimer and Au island-Au bridge-[Au@Rh(OH)_3_] dumbbell structures (Figure 2). Interestingly, by tuning the growth kinetics, Au plate-Au bridge-[Au@Rh(OH)_3_] dumbbell structures were also obtained (Figures 3, 4). With structural variations, the localized surface plasmon resonance (LSPR) and surface-enhanced Raman scattering (SERS) of the Au–Au heterostructures exhibited excellent responses, thus providing a new method for LSPRs tuning for plasmonic applications.

## 2 Experimental section

### 2.1 Materials and methods

All the chemical reagents were used as purchased without further purification. Hydrogen tetrachloroaurate (III) hydrate (HAuCl_4_·3H_2_O), 99.9% (metal basis Au 49%), trisodium citrate dihydrate (99%) was purchased from Alfa Aesar; 2-mercapto-5-benzimidazolecarboxylic acid (MBIA) (97%), rhodium (III) chloride trihydrate (98%), ascorbic acid (99%) was purchased from Aladdin; and polyvinyl pyrrolidone (average Mw 29000) was purchased from Aldrich. Absolute ethanol and deionized water (DI) (18.3 MΩ) were used for all solution preparations.

### 2.2 Characterizations

Transmission electron microscopy (TEM) images were obtained using an FEI Talos L120C microscope operated at 120 kV. Scanning electron microscopy (SEM) measurements were performed using a Quanta 250 FEG scanning electron microscope. High resolution TEM (HRTEM) was performed using a JEOL JEM-2100 transmission electron microscope operated at 200 kV. Ultraviolet-visible-near-infrared spectra were collected on a Lambda 750 ultraviolet-visible spectrophotometer. SERS was measured using a portable Raman analyzer (Accuman SR-510Pro) equipped with a 785 nm laser (350 mW) using as-synthesized sample solutions in a cuvette (1 cm optical path). The integration time was set at 10 s for all samples. X-ray photoelectron spectroscopy (XPS) spectra were collected using a Thermo SCIENTIFIC ESCALAB 250Xi photoelectron spectrometer equipped with a monochromatic Al Kα source (E = 1487.20 eV). Thermogravimetric analyses [thermal gravity (TG)/derivative thermogravimetry (DTG)] were performed using a TGA 2-1100SF thermal gravimetric analyzer.

### 2.3 Synthesis of Au–Au homometallic heterostructures

#### 2.3.1 Synthesis of Au@Rh(OH)_3_ core-shell nanoparticles

In a typical synthesis, the as-synthesized citrate-stabilized Au nanospheres (48 nm in diameter, Figure 1A) were incubated with the MBIA ligand at 60°C for 2 h. After cooling to room temperature (RT), different amounts of RhCl_3_ (10 mM) were added to the above solution under vigorous vortexing, followed by heating in an oven at 100°C for 15 min. Subsequently, 200 μl of the product was separated through centrifugation at 3,500 rpm for 10 min. The concentrated NPs collected at the bottom of the centrifuge tube were directly used in the TEM/SEM characterization. The other 500 μl of the as-synthesized sample was incubated with 2 μL of 10 mM 2-naphthalenethiol ligand at 60°C for 2 h and thereafter used for SERS measurement. The remaining 300 μl was diluted five times for absorption spectrum collection.

#### 2.3.2 Synthesis of Au Island-Au Bridge-[Au@Rh(OH)_3_] dumbbell structures

For the synthesis of Au–Au hybrid nanostructures, Au–Rh (OH)_3_ core-shell NPs with varying shell thicknesses were used as seeds, L-ascorbic acid (AA) as the reductant, and HAuCl_4_ as the precursor. In a typical synthesis, 1 mL of the Au@Rh(OH)_3_ NPs with a 12 nm Rh(OH)_3_ shell thickness was centrifuged and re-dispersed in 1 mL of water to remove residual chemicals. To prevent aggregation of the NPs during the growth process, 32 μl of polyvinylpyrrolidone (PVP, 2 mg/mL) was added as a surfactant under vigorous vortexing. After HAuCl_4_ (10 mM) was added dropwise to the solution, the mixture was incubated at RT for 1 h. Subsequently, AA (10 mM) was added in one shot under vigorous vortexing. Upon the addition of AA, the color of the solution changed immediately from red to dark purple, indicating the initiation of the growth of Au on the seed NPs. The mixture was left undisturbed for 30 min for the completion of the reactions and thereafter separated for TEM/SEM characterization.

#### 2.3.3 Synthesis of Au Island-on-[Au@Rh(OH)_3_] structures

The synthesis of Au island-on-[Au@Rh(OH)_3_] structures followed the same procedure as that for the synthesis of Au island-Au bridge-[Au@Rh(OH)_3_] dumbbell structures but without pre-incubation of the Au@Rh(OH)_3_ seeds and the HAuCl_4_ precursor.

## 3 Results and discussion

For the synthesis of the Au–Au heterostructures, seeds of Au@Rh(OH)_3_ core-shell structures with varying Rh(OH)_3_ shell thicknesses were synthesized *via* the seed-mediated growth of Rh(OH)_3_ on citrate-stabilized AuNPs (Figure 1, Supplementary Figures S1, S2).

As shown in Figures 1B–F, with an increase in the RhCl_3_ and MBIA ligand (50–200 μM, Supplementary Table S1), concentric Au@Rh(OH)_3_ core-shell structures with increasing Rh(OH)_3_ shell thickness (1.5–18 nm) were obtained. From the enlarged TEM images, a uniform Rh(OH)_3_ layer can be observed on the surface of the Au seeds, even with a very thin thickness (1.5 and 2.5 nm in Figures 1B, C). From the inset photographs, the color of the Au@Rh(OH)_3_ samples changed from red to dark red and brown with an increase in the shell thickness (Figures 1A–F). The absorption peaks of the Au cores in Au@Rh(OH)_3_ were continuously redshifted from 540 nm (Au seeds) to 544, 547, 553, 563, and 579 nm (Figure 1G). This can be attributed to the increase in the refractive index owing to the increase in the Rh(OH)_3_ shell thickness (Yue et al., 2016; Xu et al., 2021).

The synthesis of Au@metal oxide core-shell structures has been extensively studied (Sun et al., 2013; Lukosi et al., 2016). However, reports on Au@metal hydroxide core-shell structures are rare because of the low compatibility and significant mismatch between Au and hydroxide materials (Zhou and Zeng, 2016; Cai et al., 2020).

In our synthesis, the Au seeds were preincubated with MBIA ligands at 60°C for 2 h prior to the growth of the Rh(OH)_3_ shell. The MBIA ligand played dual roles in the formation of Au@Rh(OH)_3_ core-shell NPs: 1) Modification of Au seeds with a stable MBIA layer through the strong interaction of the -SH group with Au (Feng et al., 2012). The control experiment demonstrated that without the pre-incubation of Au seeds with the MBIA ligand, a mixture of free Rh(OH)_3_ NPs and Au seeds was observed (Supplementary Figure S3). This revealed that citrate-stabilized AuNPs are not suitable for the heterogeneous nucleation and growth of Rh(OH)_3_ because of the high interfacial energy (the difference in surface energy, Peng and Yang, 2009; Carbone and Cozzoli, 2010) between the Au and Rh(OH)_3_ materials. For Au@MBIA seeds, the dense -COOH groups on the surface can act as anchors for the partially hydrolyzed Rh^3+^ cations through the formation of the COO-Rh(OH)_n_ (*n* < 3) complex (Kataoka et al., 2010; Enriquez Garcia and Jalilehvand, 2018), promoting the formation of Rh(OH)_3_ shells on Au seeds. 2) The formation of the Rh^3+^-MBIA complex in the growth solution played a vital role in the formation of the Rh(OH)_3_ shell on the Au seeds by slowing the hydrolysis rate of Rh^3+^ ions.

XPS, TG, and DTG measurements were performed to confirm the composition of the shell material. As shown in the XPS spectrum (Figure 1), the two peaks observed at 309.4 and 314 eV can be assigned to the Rh 3d_5/2_ and Rh 3d_3/2_ peaks, respectively, confirming the +3 valency of the Rh cation (Kim and Hatfield, 1991). From the TG/DTG analysis (Figure 1H), the first mass loss (4.5%) at temperatures less than 325°C can be assigned to the loss of crystal water. The second mass loss (8.5%) in the temperature range of 325°C–350°C was due to the dehydration of Au@Rh(OH)_3_, from which the shell was confirmed as Rh(OH)_3_ (Schünemann et al., 1994).

For the synthesis of Au–Au hybrid nanostructures, Au–Rh(OH)_3_ core-shell NPs with varying shell thicknesses were used as seeds, AA as the reductant, and HAuCl_4_ as the precursor (Figure 2). In a typical synthesis, the Au@Rh(OH)_3_ NPs were centrifuged and redispersed in the same volume of DI water, followed by the addition of PVP to prevent the aggregation of the NPs during growth. After HAuCl_4_ (10 mM) was added dropwise to the above solution, the mixture was incubated at RT for 1 h and thereafter, AA was added in one shot under vigorous vortexing. The mixture was left undisturbed for 30 min to complete the reaction.

As shown in Figure 2B, when Au@Rh(OH)_3_ NPs with a shell thickness of 12 nm were used as seeds, Au island-Au@Rh(OH)_3_ dimers were obtained. Interestingly, a thin Au bridge (approximately 12 nm in length and 6 nm in diameter) in the Rh(OH)_3_ shell can be observed in 57% of the NPs (Figure 2C; Supplementary Figure S4), linking the Au core and the outer Au island to Au island-Au bridge-[Au@Rh(OH)_3_] dumbbell structures with a thin “handle”. Such dumbbell-shaped structures were achieved only using the physical lithography method on a solid substrate. Colloidal synthesis remains a significant challenge due to the lack of an effective control method (Koya and Lin, 2016; Wang et al., 2019a).

Because the Au island-Au bridge-[Au@Rh(OH)_3_] dumbbell structures were grown from Au@Rh(OH)_3_ seeds, it can be concluded that the growth startarted from the nucleation and growth of the Au bridge on the surface of the Au core in the Rh(OH)_3_ shells. When the growth of the Au bridge penetrated the Rh(OH)_3_ shells, an Au island formed at the end. As shown in Figure 2D, for Au@ Rh(OH)_3_ seeds, a single absorption peak was observed at 560 nm. After the growth of the Au islands, except for the redshift of the 560 nm peak, new broad absorptions were observed at 650 and 700 nm for structures 2B and 2C, respectively. Considering the high similarity in structural parameters for structures 2B and 2C, their variance in absorption spectra should arise from the different plasmonic couplings with and without the thin Au bridge between the Au core and newly grown Au islands (Wang et al., 2017; Zhou et al., 2018; Dhiman et al., 2019).

Similar to the inorganic oxide polymer SiO_2_ or TiO_2_ (Song et al., 2015; Yu et al., 2021), the Rh(OH)_3_ shell condensed from the hydrolysis of RhCl_3_ in a basic aqueous solution should be highly porous (Supplementary Figure S5). When incubating with HAuCl_4_, the AuCl_4_^−^ ions diffused into the pores of the Rh(OH)_3_ shells.

It is known that the heterogeneous nucleation would occur prior the homogeneous nucleation due to its lower nucleation barrier (Wang et al., 2015). For the Au@Rh(OH)_3_ seeds, there are two types of sites for the heterogeneous nucleation of Au: the Au cores in the Rh(OH)_3_ shells and the surface of the Rh(OH)_3_ shells. From the perspective of interfacial energy, the Au core should be the preferential site for the same Au material, and the surface of the Rh(OH)_3_ shell should have a higher nucleation barrier. This variance can be reflected at different critical growth material concentrations (CGMC) needed for nucleation, that is, lower CGMC on the Au core and higher CGMC on the Rh(OH)_3_ shell (Wang et al., 2015).

Upon the addition of the reductant AA, Au atoms can be produced where AA reaches *via* diffusion both in the growth solution and inside the pores. Therefore, the concentration of Au atoms in the solution should be higher than that in the Rh(OH)_3_ shells as the slower diffusion of AA inside the Rh(OH)_3_ shells. However, the lower nucleation barrier resulted in a lower CGMC for the nucleation on the inner Au core. Thus, the heterogeneous nucleation of Au on the Au core can occur prior to the outer Rh(OH)_3_ shells.

Owing to the MBIA ligand layer on the Au cores, single-site nucleation of Au occurred because of the high interfacial energy between the Au@MBIA and Au deposit (Feng et al., 2012). According to the depletion sphere model (Feng et al., 2017), the formation of a nucleus depletes Au atoms (both inside and outside the Rh(OH)_3_ shells). The low concentration of Au atoms (<CGMC) cannot support the formation of a new nucleus nearby. This is the reason why only one Au bridge on each AuRh(OH)_3_ seed is the dominant product.

Determined by the diffusion direction of AA from the solution vertical to the Au cores, the extrusion growth of Au in the Rh(OH)_3_ shell leads to the formation of a thin Au bridge vertical to the seed surface. When the growth of the Au bridge penetrated the Rh(OH)_3_ shell, the end of the Au bridge grew into a spherical Au island because of the fast growth under high concentrations of Au atoms in the growth solution.

To confirm the above hypothesis, control experiments were performed under similar conditions, but without the pre-incubation of Au@Rh(OH)_3_ seeds and HAuCl_4_. As shown in Figure 2B, all the Au islands grew on the surface of the Rh(OH)_3_ shells, forming Au island-on-[Au@Rh(OH)_3_] structures. This result demonstrates that the pre-incubation of Au@Rh(OH)_3_ and HAuCl_4_ is the key step for the formation of a thin Au bridge. In contrast to the 1:1 Au island-Au bridge-[Au@Rh(OH)_3_] structures, two to three Au islands grew on each Au@Rh(OH)_3_ seed. This multi-island growth of Au on the Au@Rh(OH)_3_ seeds likely resulted from the fast nucleation at high Au atom concentrations (Feng et al., 2017). Without preincubation, AA was added immediately after the addition of HAuCl_4_. Without mass loss due to diffusion into the Rh(OH)_3_ shells, the reduction of HAuCl_4_ at high concentrations led to increased oversaturation of Au atoms at the moment of nucleation. Thus, multi-site nucleation can occur. Based on the above conditions, when the concentration of AA was reduced from 0.7 to 0.4 mM, single-island growth of Au on the surface of the Au@Rh(OH)_3_ seeds was observed (Figure 3A), confirming the reduction rate effect during nucleation.

With pre-incubation, a lower [AA] led to the formation of Au island-Au@Rh(OH)_3_ (80%) and Au plate-Au@Rh(OH)_3_ dimers (20%) (Figure 3B). The enlarged TEM images (Supplementary Figure S6) demonstrate that the Au island domains in the Au island-Au@Rh(OH)_3_ dimers are thick Au bridges grown on the Au core. Because the surface area of the end of the Au bridge is very large, when it penetrates the Rh(OH)_3_ shell, the insufficient supply of Au atoms cannot support the formation of Au islands. Therefore, unlike the thin Au bridge, no Au islands are formed at the end of the Au bridge. As shown in Figure 3C, a single broad absorption in the wavelength range of 500–800 nm was observed for both structures 3A and 3B. In comparison to structure 3A, the slight redshift and clear broadening of the absorption for structure 3B may be due to the formation of more new Au domains in plate shapes. It should be noted that due to the relative low uniformity of the Au-Au structures, the measured absorption is the ensembled average absorptions of the mixed structures, which cannot be precisely assigned in analysis.

It is well known that the synergistic effect of the presence of the twin plane and selective ligand blocking of the Au 111 surface is responsible for the formation of the Au nanoplate. PVP is known as a surface-blocking agent for the synthesis of Au nanoplates because of its strong coordination on the Au 111 surface (Wang et al., 2010; Wang et al., 2016; Balasubramanian and Raghavachari, 2017). Thus, we speculate that the coexistence of the Au island-Au@Rh(OH)_3_ and Au plate-Au@Rh(OH)_3_ structures is due to the random twinning during the growth of the Au bridges. The Au bridges without twin planes grew into Au islands (thick bridges), whereas those with twin planes vertical to the surface of the Au cores grew into Au nanoplates after they penetrated the Rh(OH)_3_ shells. As shown in Figure 3B, the size of the Au plates is significantly larger than that of the Au islands in Au–Au dimers. This was due to the fast growth of Au at the concave defect site of the twin plane (Jiang et al., 2018).

Interestingly, when Au@Rh(OH)_3_ seeds with thinner shells (8 nm) were pre-incubated with HAuCl_4_, the growth of Au nanoplates with higher purity (approximately 33%) was observed. At a lower AA (0.4 mM), the purity of the Au plate-Au bridge-[Au@Rh(OH)_3_] structure was further increased to 37% (Figures 4A, B). When the thickness of the Rh(OH)_3_ shell was further decreased to 5 nm and 2.5 nm, the purity of Au plate-Au bridge-[Au@Rh(OH)_3_] structures continuously increased slightly (40% and 44%, respectively). From the absorption spectra shown in Figure 4E, with the decrease in the Rh(OH)_3_ shells, the transverse absorption of Au continuously blue-shifted from 563 to 560, 554, and 553 nm owing to the decrease in the refractions of Rh(OH)_3_. In addition, with an increase in the plate-shaped Au domains, the longitudinal absorptions for structures A–D changed from shoulder peaks (A and B) to single broad absorptions. The continuous redshift (from 638 to 643, 738, and 766 nm) was a result of the increase in the structural anisotropy and LSPRs coupling between the Au core and outer Au nanoplates (Feng and Chen, 2021).

When the shell thickness was greater than 12 nm, the Au island-Au bridge-[Au@Rh(OH)_3_] structure became the dominant product. In addition, the yield decreased with increasing shell thickness (Supplementary Figure S7). A possible reason for this observation is that because of the longer diffusion path of AA from the solution to the inner Au core, the nucleation of Au on the surface of the Rh(OH)_3_ shell may occur first, leading to the formation of the Au island-on-[Au@Rh(OH)_3_] structure. The thicker the Rh(OH)_3_ shell, the higher the yield of the Au island-on-[Au@Rh(OH)_3_] structure (Supplementary Figure S7).

**FIGURE 5:**
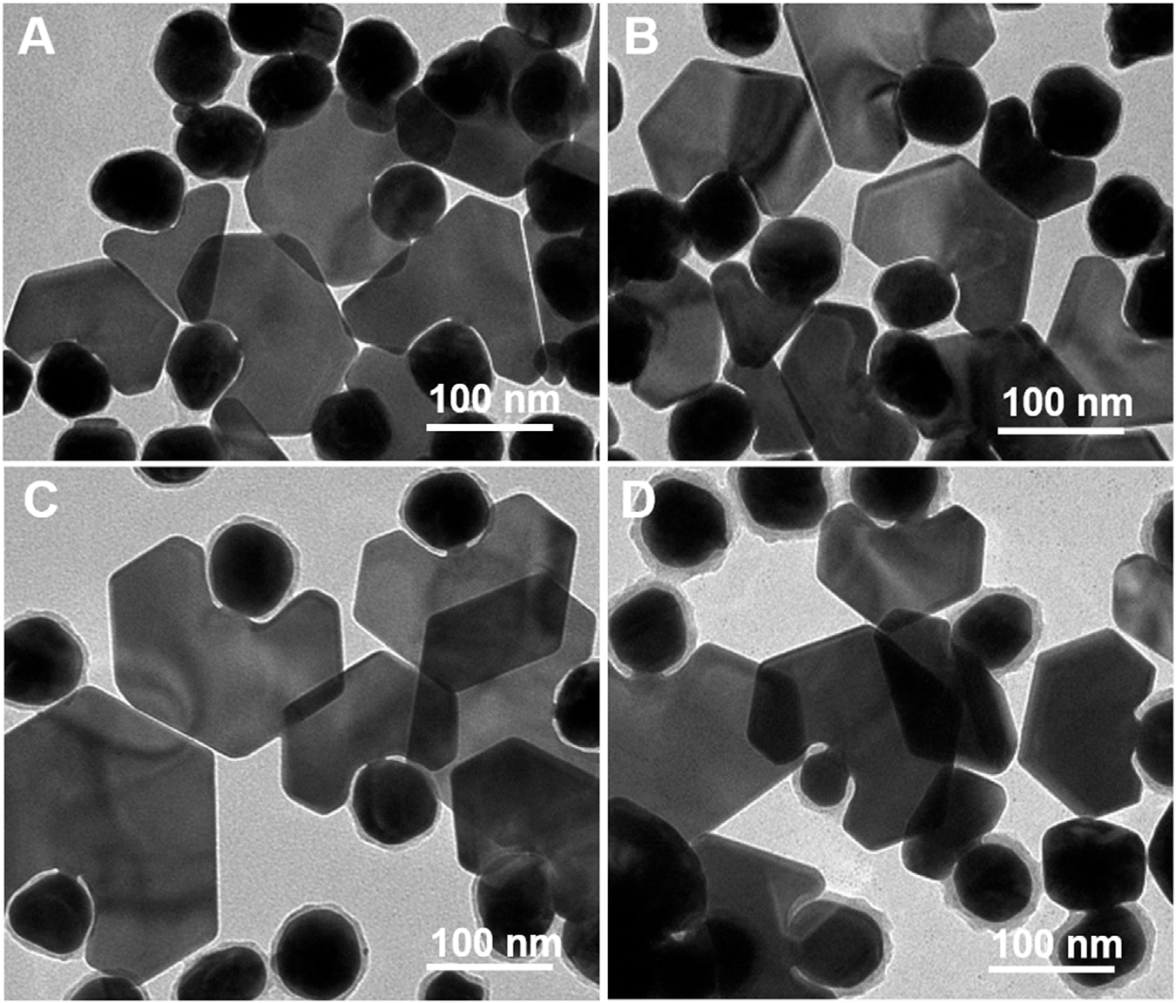
TEM images of the Au plate-Au bridge-Au@Rh(OH)_3_ structures with different size of Au bridges synthesized using Au@Rh(OH)_3_ seeds with varying shell thickness: **(A)** 2.5, **(B)** 5, **(C)** 8, and **(D)** 12 nm.

When the shell thickness is different, except for the different purities of the products, the sizes of the Au bridges also differ. The enlarged TEM images illustrated in Figure 5 show that when the thickness of the Rh(OH)_3_ shell increased from 2.5 to 5, 8, and 12 nm, the length of the Au bridges increased from 2.5 to 3.5, 5.5, and 8 nm, and the width increased from 3 to 7, 8.5, and 12 nm, respectively. It is reasonable to assume that the length of the Au bridges is determined by the thickness of the Rh(OH)_3_ shell. The width of Au bridges is highly dependent on their length. Generally, the growth of an Au bridge in the vertical and lateral directions occurs simultaneously. With an increase in length, the width also continuously increased owing to the longer growth time. From the HRTEM analysis, the Au plate and Au bridge are epitaxially grown from the surface of the Au seeds (Supplementary Figure S9).

## 4 Conclusion

In conclusion, we developed a new method for synthesizing bridged Au–Au dumbbell structures by controlling the diffusion of the HAuCl_4_ precursor in the porous Rh(OH)_3_ shell of Au@Rh(OH)_3_ seeds. While the pre-incubation of the Au@Rh(OH)_3_ seeds and HAuCl_4_ is a prerequisite for the formation of Au bridges in Rh(OH)_3_ shells, the thickness of the Rh(OH)_3_ shells and the concentration of AA reductant can both affect the formation of Au bridges and the shape of the end Au domains, respectively. Importantly, the growth of the Au bridge was completely different from the conventional wetting growth of Au on Au seeds. In the porous Rh(OH)_3_ shells, the extrusion growth of Au inhibited their wetting of Au seeds. We believe that this new diffusion control approach will create new approaches for achieving site-specificity in colloidal synthesis, which would eventually promote the synthesis of new sophisticated functional nanomaterials.

## Data Availability

The original contributions presented in the study are included in the article/Supplementary Materials, further inquiries can be directed to the corresponding authors.
