# Supplementary material for: Diffusion-controlled bridging of the Au Island and Au core in Au@Rh(OH)3 core-shell structure
Source: Front Chem. 2023 Jan 25;11:1138932. doi: 10.3389/fchem.2023.1138932 (PMC9905440; doi:10.3389/fchem.2023.1138932)
Supplement: Supplementary file 1 [file DataSheet1.docx]

Supplementary Material

Diffusion-controlled Bridging of the Au Island and Au Core in Au@Rh(OH)_3_ Core-shell Structure

Jie Zhang^1, †^, Quan Ren^1, †^, Yun Wang^1^, Ruixue Xiao^1^, Hongyu Chen^1,3^, Wenjia Xu^2,^*, and Yuhua Feng^1,^*

^1^Institute of Advanced Synthesis, School of Chemistry and Molecular Engineering, Nanjing Tech University, Nanjing 211816, China

^2^School of Physical and Mathematical Science, Nanjing Tech University, Nanjing 211816, China

^3^School of Science, Westlake University, Hangzhou 310023, China

^†^ These authors contribute equally to this work

*** Correspondence:**
Wenjia Xu

[xwj0729@126.com](mailto:xwj0729@126.com)

Yuhua Feng
[ias_yhfeng@njtech.edu.cn](mailto:ias_yhfeng@njtech.edu.cn)

**Synthesis of Au nanoparticles:**

Step 1: Seed preparation.

Adding 25 mL ultra-pure water to a 500 mL flask, then add 0.25 mL (10 mg/mL) HAuCl_4_ solution to it, followed by stirring at high speed in an oil bath at 135 °C for 30 min, then adding 0.375 mL (10 mg/mL) sodium citrate to the flask under intense stirring, the solution color changed from colorless to black, then to purple, and finally to red. After the color is stable, the size of the gold nanoparticles is about 40 nm after reflux for 30 min.

Step 2: Multi-step growth.

After reflux for 30 min, 25 mL hot ultra-pure water, 0.05 mL (6.6 mg /mL) NaOH, 0.25 mL (10 mg/mL) sodium citrate and 0.25 mL (10 mg/mL) HAuCl_4_ were quickly added successively. After refluxing for 30 min, 50 mL hot ultra-pure water, 0.1mL (6.6 mg /mL) NaOH, 0.5 mL (10 mg/mL) sodium citrate and 0.25 mL (10 mg/mL) HAuCl_4_ were added in rapid order for 30 min (repeat this step twice), until the above steps were completed. Add 0.1 mL (6.6 mg/mL) NaOH, 0.5 mL (10 mg/mL) sodium citrate and 0.25 mL (10 mg/mL) HAuCl_4_, and reflux for 30 min (repeat this step twice). Finally, the solution was cooled at room temperature for 12 h before using.


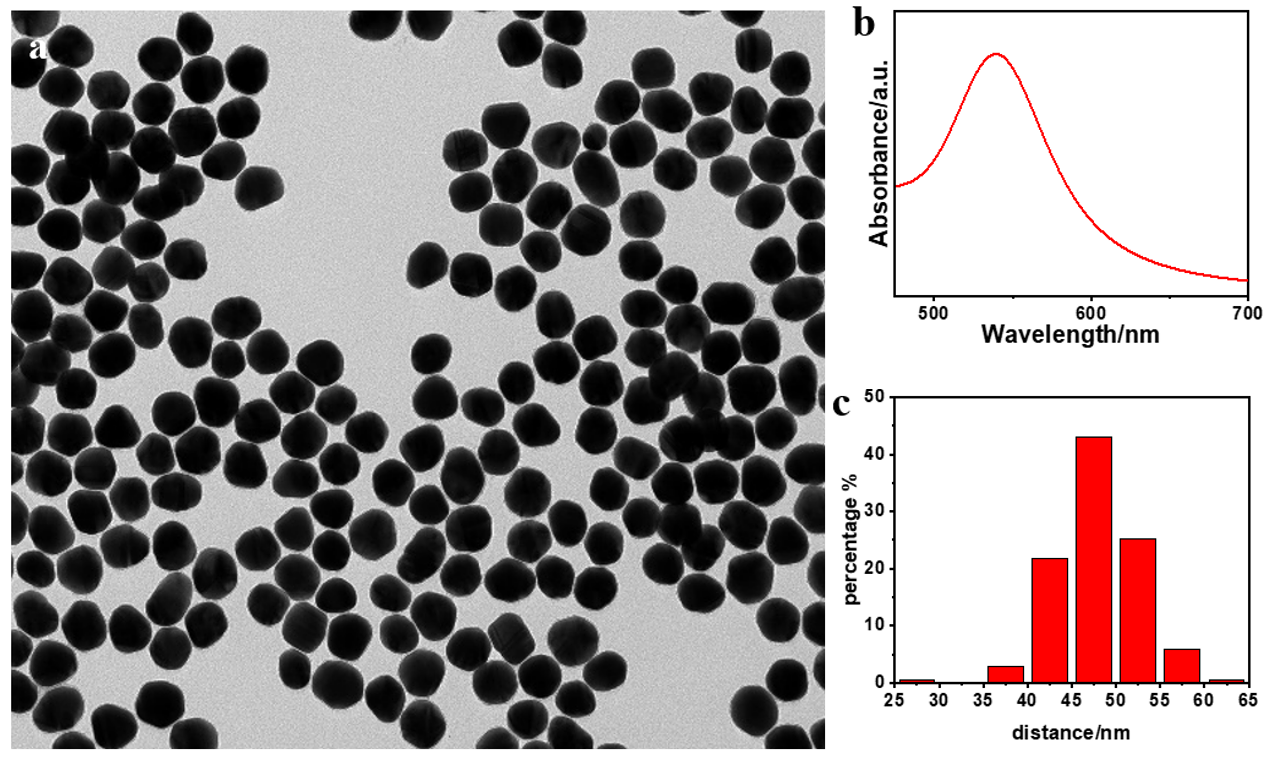


**FIGURE S1 |** (a) TEM image, (b) absorption spectrum and (c) size distribution of the Au seeds.

**TABLE** **S1 |** Synthetic conditions for Au@Rh(OH)_3_ core-shell NPs with different shell thickness

| Shell thickness/nm | MBIA/μM | RhCl_3_/μM |
| --- | --- | --- |
| 1.5 | 50 | 50 |
| 2.5 | 50 | 100 |
| 5 | 100 | 100 |
| 8 | 100 | 150 |
| 12 | 150 | 150 |
| 14 | 200 | 200 |
| 18 | 200 | 250 |


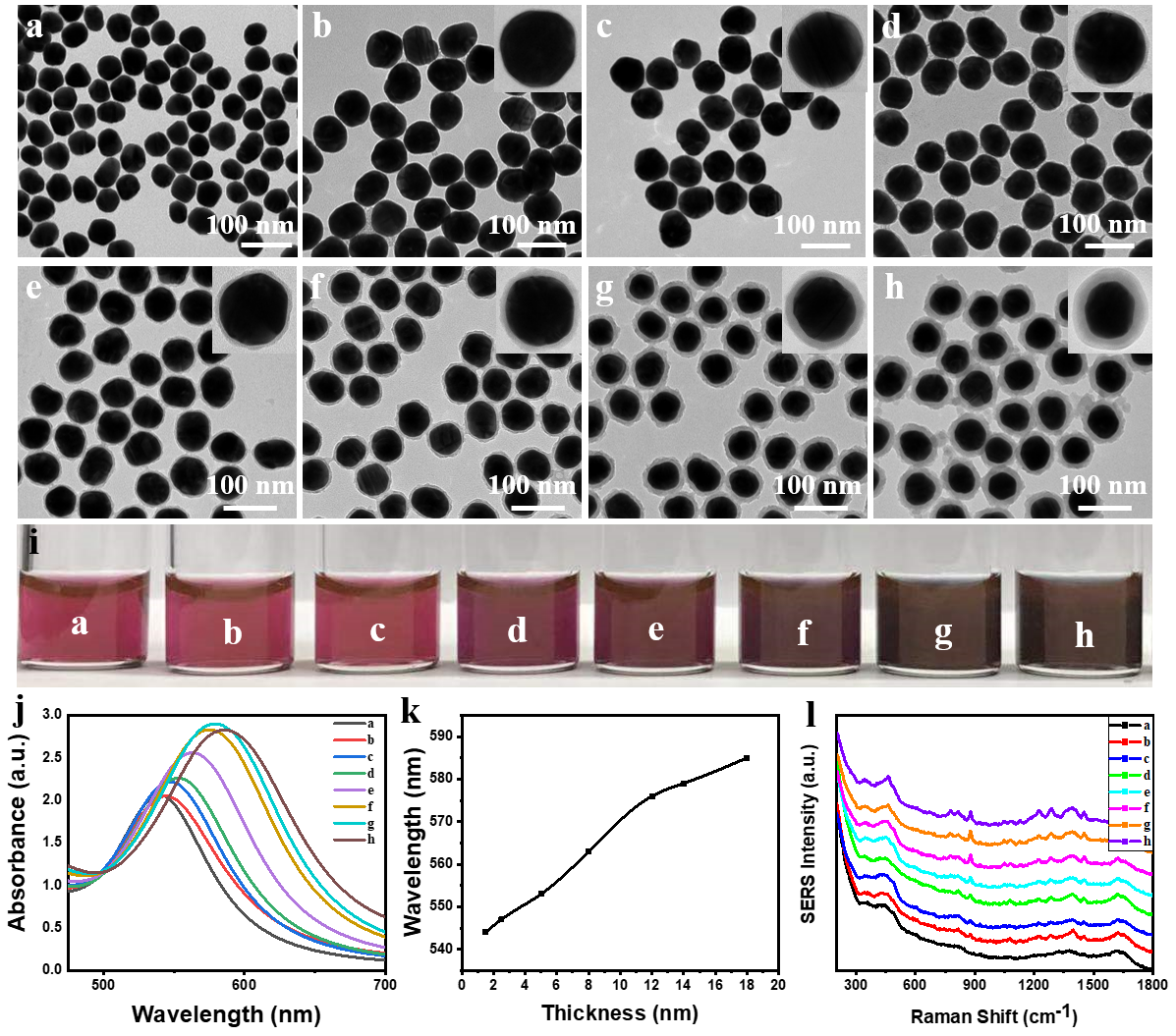


**FIGURE S2 |** (a-h) TEM images and photographs of Au seed and Au@Rh(OH)_3_ core-shell structures with different shell thickness. (j) Absorption spectra of the Au@Rh(OH)_3_ NPs, (k). Plot of the shell thickness and the maximum absorption of Au@Rh(OH)_3_ NPs, (l) SERS of Au@Rh(OH)_3_ with different shell thickness. The weak Raman shifts are arisen from the MBIA ligand at Au-Rh(OH)_3_ interface.


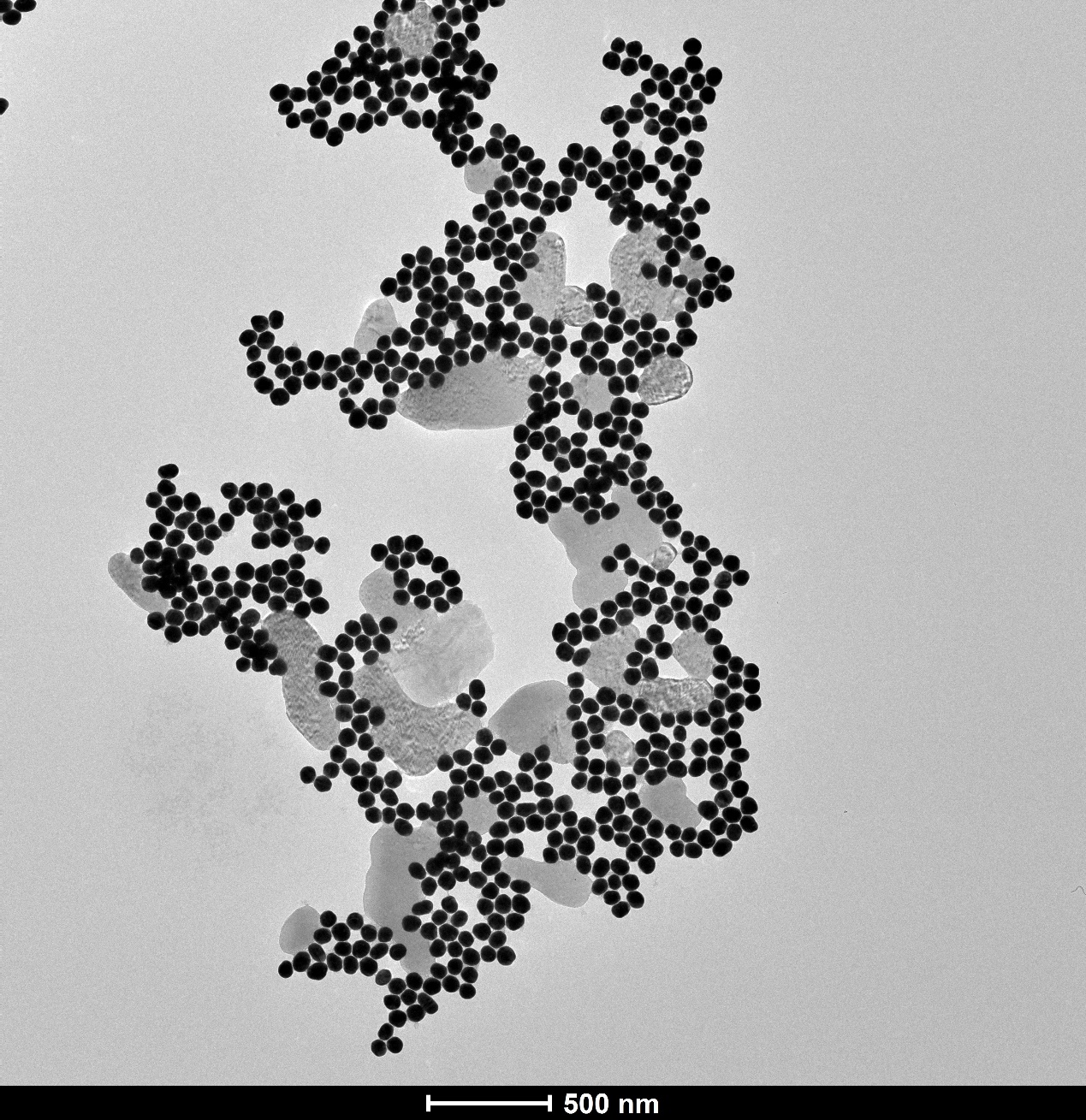


**FIGURE S3 |** In the absence of pre-incubation of Au seeds with MBIA, bare AuNPs and free Rh(OH)_3_ NPs were obtained.


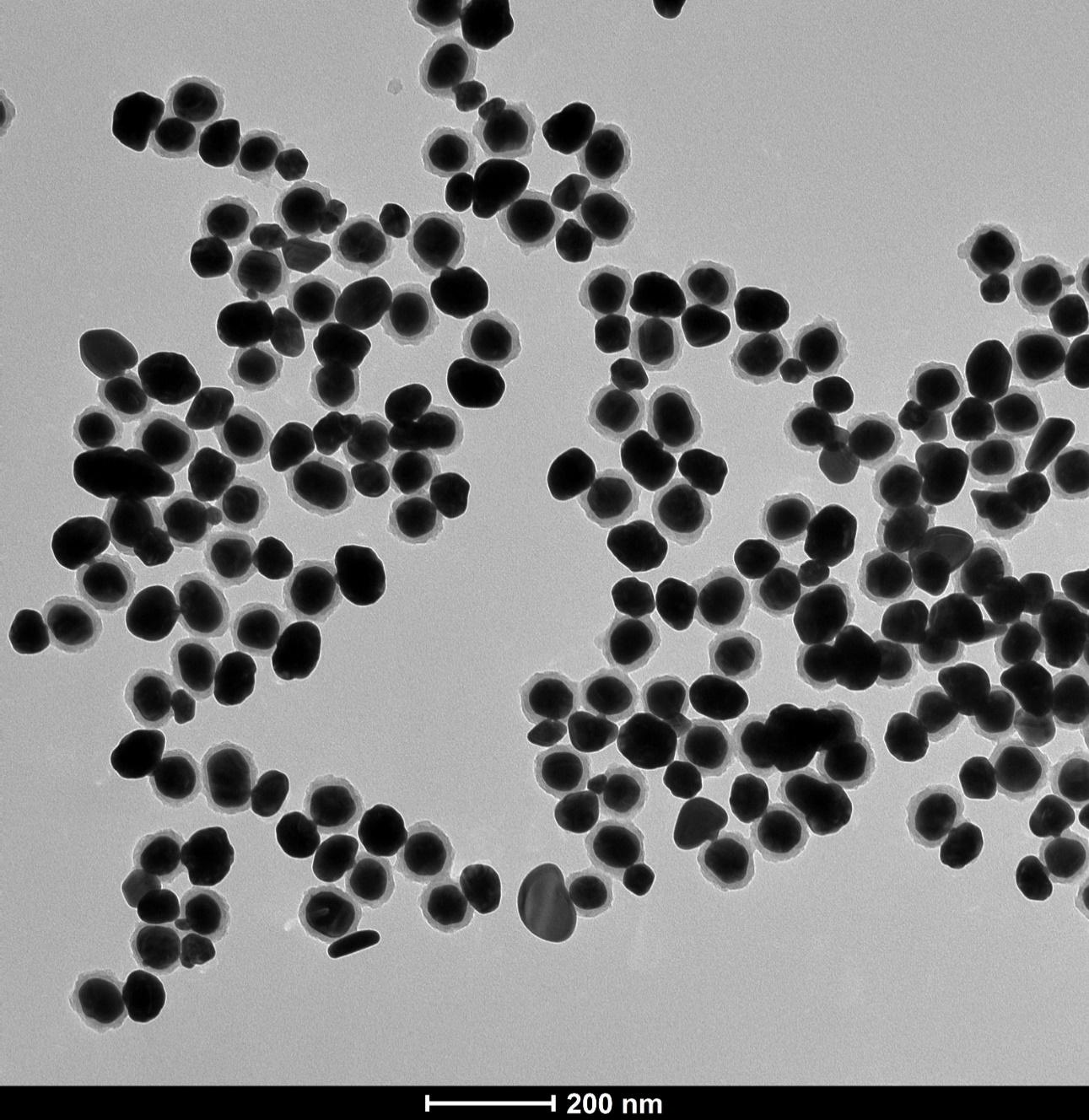


**FIGURE S4 |** The original image of **Figure 2C**.


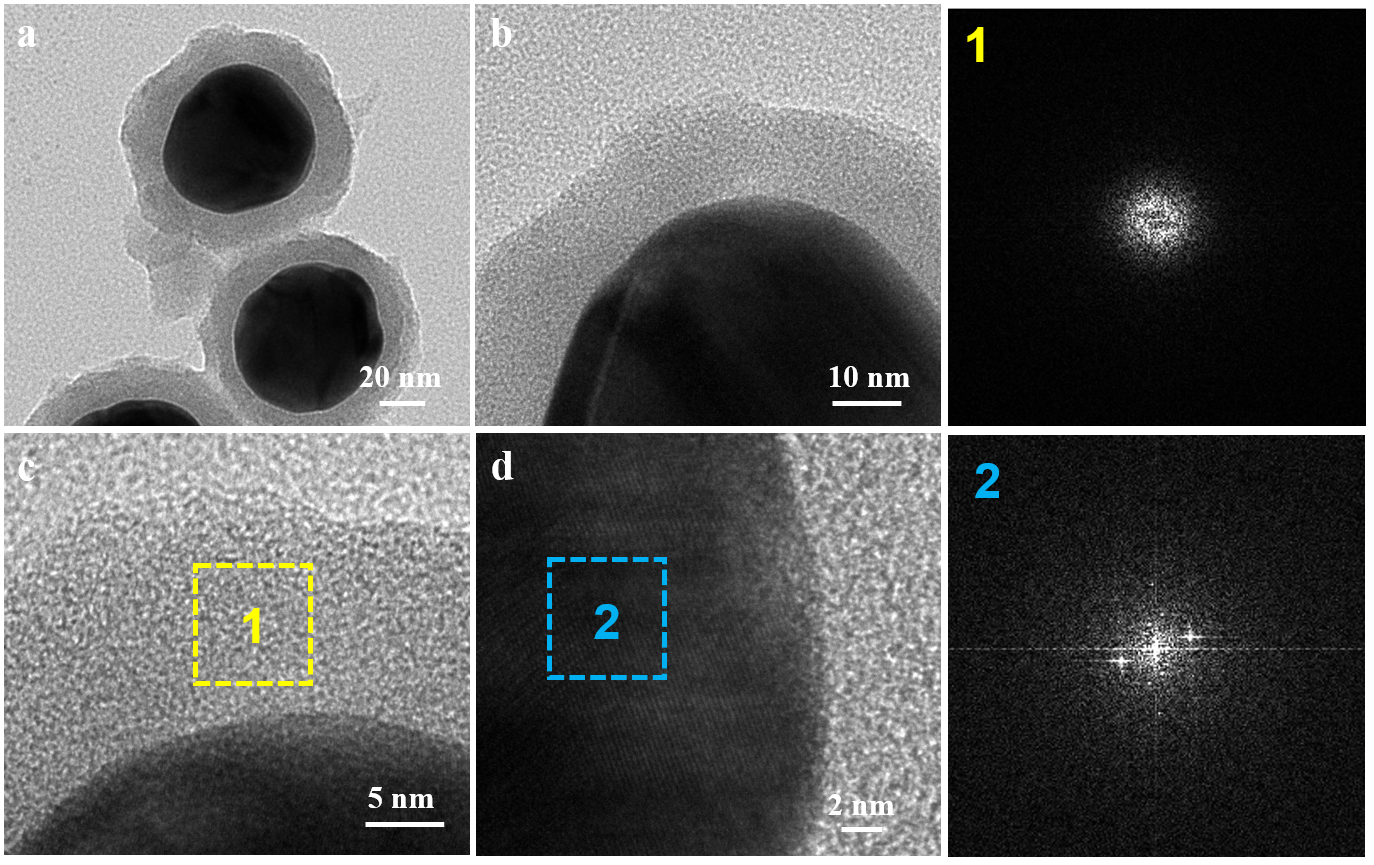


**FIGURE S5 |** (a-d) HRTEM of an Au@Rh(OH)_3_ structure.

The corresponding Fast Fourier transform (FFT) pattern shows only a weak reflection halo with no diffraction spots, suggesting their amorphous feature (Ge et al., 2020).


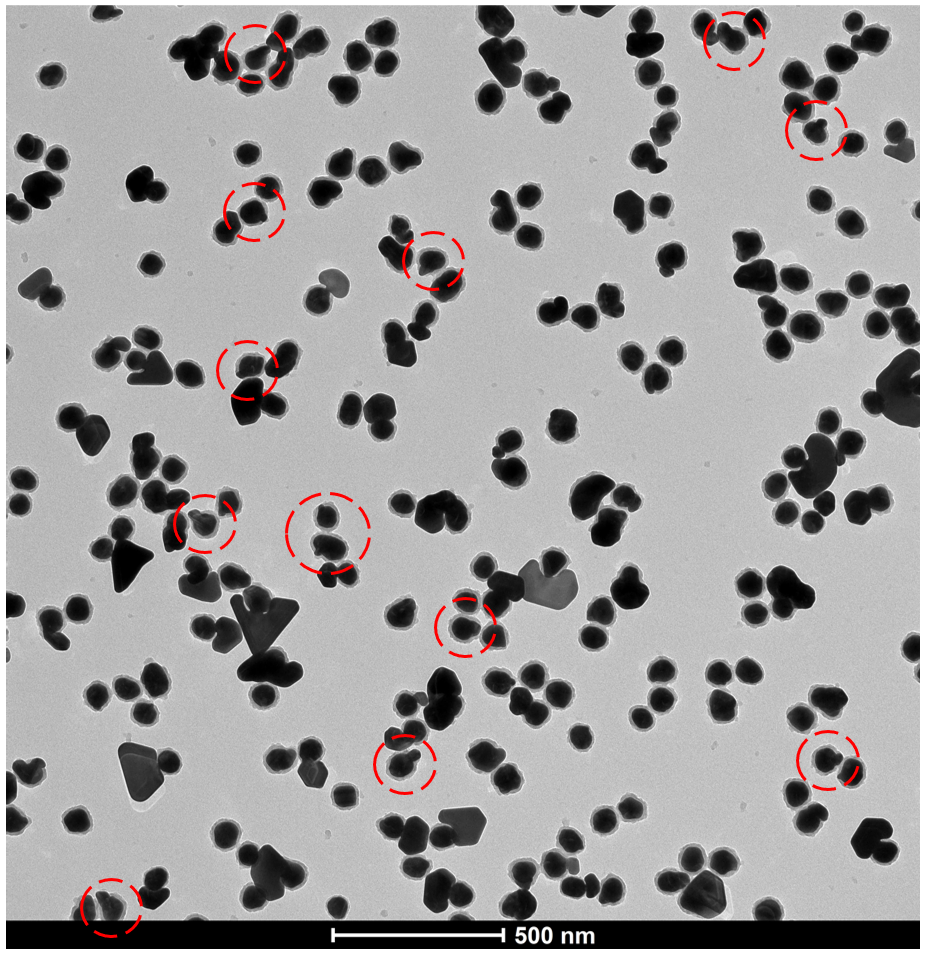


**FIGURE S6 |** The Au island domains in Au island-Au@Rh(OH)_3_ dimers are thick Au bridge grown on the Au core.


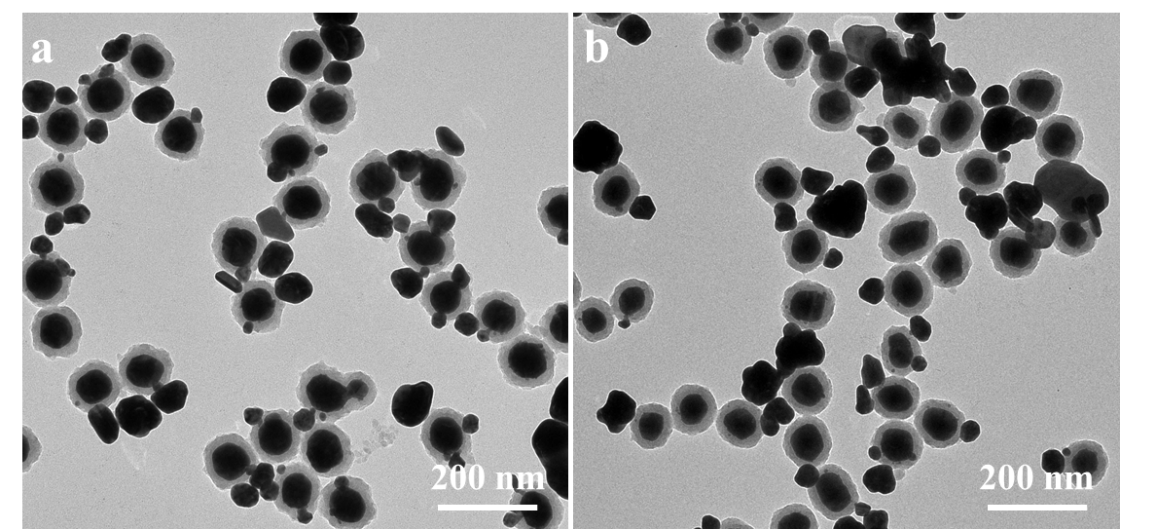


**FIGURE S7 |** TEM images of Au-Au@Rh(OH)_3_ structures synthesized by using Au@Rh(OH)_3_ seeds with 18 nm shell thickness, (a) with, and (b) without pre-incubation of Au@Rh(OH)_3_ and HAuCl_4_.


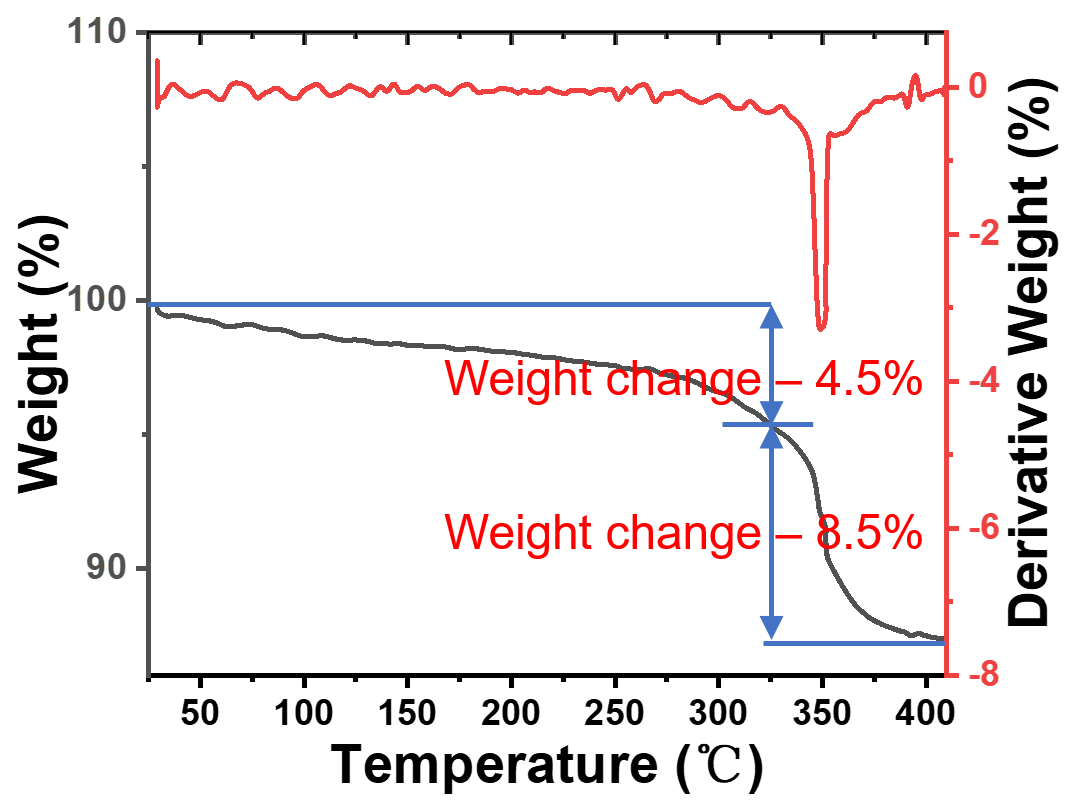


**FIGURE S8 |** Analysis of TG/DTG.


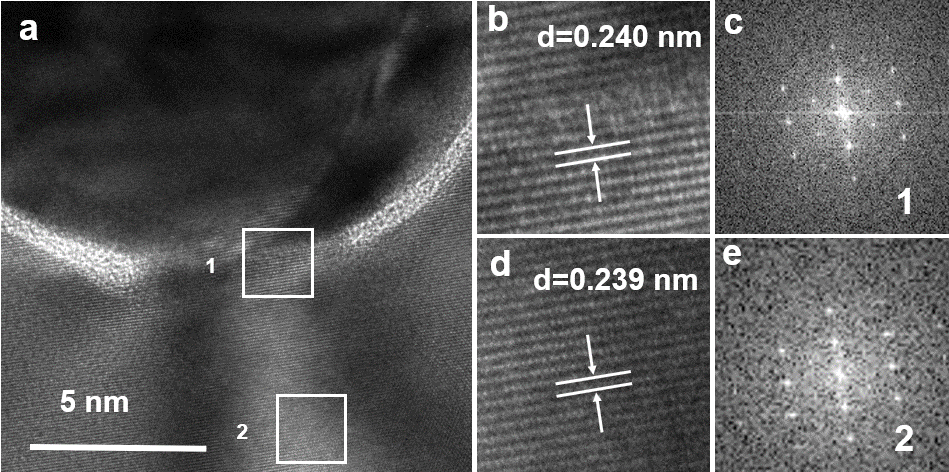


**FIGURE S9** | (a) HRTEM of an individual Au plate-Au-bridge-[Au@Rh(OH)_3_] nanoparticle. (b-e) Enlarged lattice and FFT pattern of the selected area of 1 and 2 squared in (a).

For the Au plate-Au-bridge-[Au@Rh(OH)_3_] structure, the HRTEM analysis showed epitaxial growth of the Au bridge and plate on Au seeds. As shown in Figure S9b and d, the lattice fringe of the squared area 1 (the Au bridge) and 2 (Au plate) are highly ordered, indicating both the epitaxial growth of Au bridge on Au seed and the epitaxial growth of Au plate on Au bridge. From the measured lattice spacing of 0.24 nm in area 1 and 2, the bridge and the surface of plate are assigned as Au (111) facets(Ye et al., 2019). In addition, the same orientation in FFT patterns of area 1 and 2 provided supplementary evidence for the epitaxial growth of Au nanoplate from the Au bridge (Figure S9c and e).


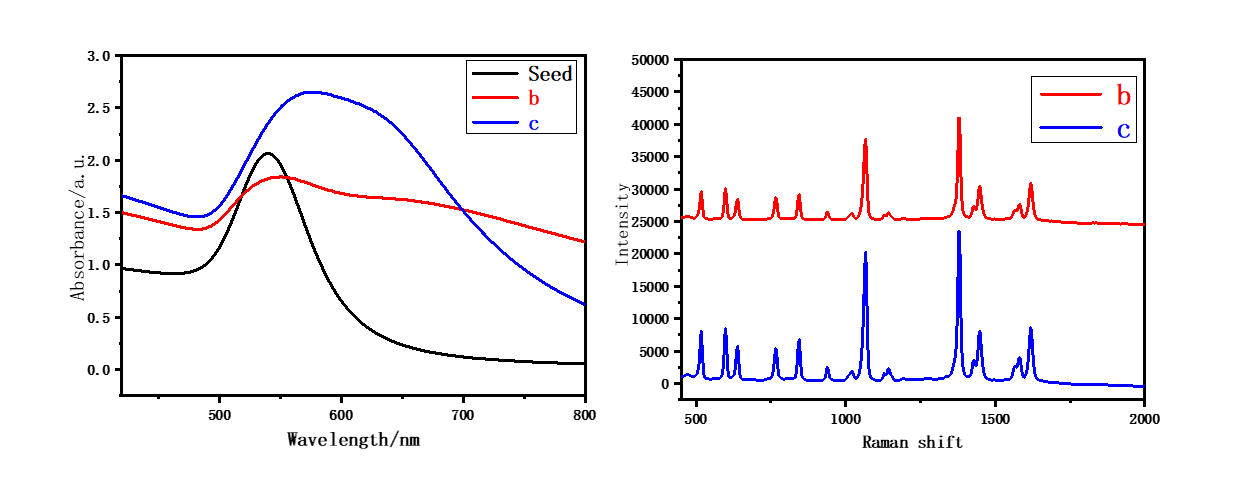


**FIGURE S10 |** SERS for the sample 2B and 2C in **Figure 2** in the main text.


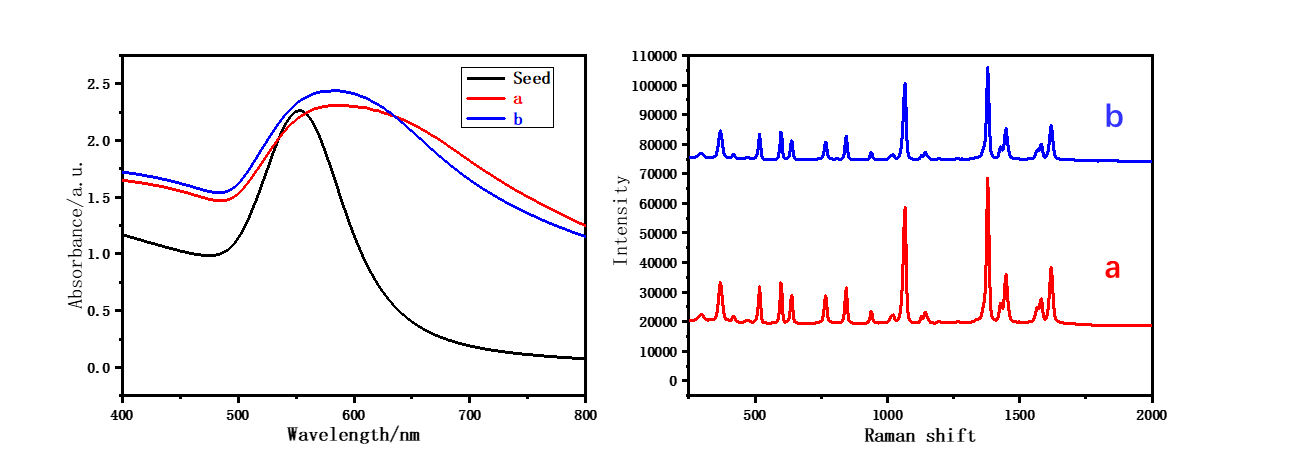


**FIGURE S11 |** SERS for the structures in **Figure 3A** and **3B** in the main text.


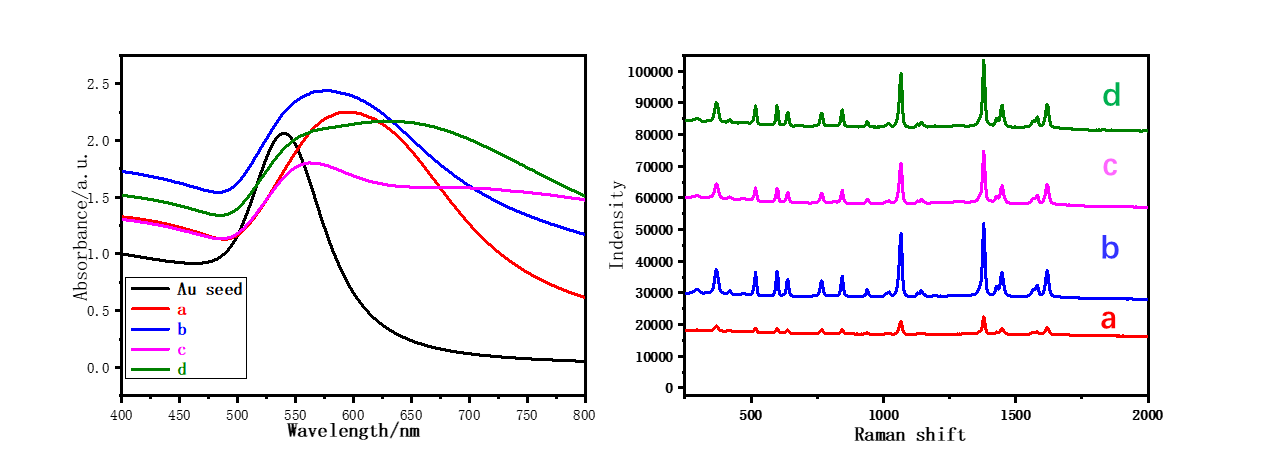


**FIGURE S12 |** SERS for the structures in **Figure 4A-D** in the main text.

**Reference:**

Ge, Y., Shi, Z., Tan, C., Chen, Y., Cheng, H., He, Q., et al. (2020). Two-Dimensional Nanomaterials with Unconventional Phases. *Chem* 6(6)**,** 1237-1253. doi: 10.1016/j.chempr.2020.04.004.

Ye, S., Brown, A.P., Stammers, A.C., Thomson, N.H., Wen, J., Roach, L., et al. (2019). Sub-Nanometer Thick Gold Nanosheets as Highly Efficient Catalysts. 6(21)**,** 1900911. doi: 10.1002/advs.201900911.
